# Bacterial Cellulose for Scalable and Sustainable Bio-Gels in the Circular Economy

**DOI:** 10.3390/gels11040262

**Published:** 2025-04-02

**Authors:** Giovanni Venturelli, Federica Villa, Mariagioia Petraretti, Giuseppe Guagliano, Marinella Levi, Paola Petrini

**Affiliations:** 1 Department of Chemistry, Materials and Chemical Engineering “Giulio Natta”, Politecnico di Milano, Piazza Leonardo da Vinci 32, 20133 Milan, Italy; giovanni.venturelli@polimi.it (G.V.); giuseppe.guagliano@polimi.it (G.G.); marinella.levi@polimi.it (M.L.); 2 Department of Food, Environmental and Nutritional Sciences, University of Milan, Via Mangiagalli 25, 20133 Milan, Italy; federica.villa@unimi.it (F.V.); mariagioia.petraretti@unimi.it (M.P.)

**Keywords:** cellulose, sustainable fashion, food waste, green manufacturing, fermentation

## Abstract

Microbial-derived materials are emerging for applications in biomedicine, sensors, food, cosmetics, construction, and fashion. They offer considerable structural properties and process reproducibility compared to other bio-based materials. However, challenges related to efficient and sustainable large-scale production of microbial-derived materials must be addressed to exploit their potential fully. This review analyzes the synergistic contribution of circular, sustainable, and biotechnological approaches to enhance bacterial cellulose (BC) production and fine-tune its physico-chemical properties. BC was chosen as an ideal example due to its mechanical strength and chemical stability, making it promising for industrial applications. The review discusses upcycling strategies that utilize waste for microbial fermentation, simultaneously boosting BC production. Additionally, biotechnology techniques are identified as key to enhance BC yield and tailor its physico-chemical properties. Among the different areas where cellulose-based materials are employed, BC shows promise for mitigating the environmental impact of the garment industry. The review emphasizes that integrating circular and biotechnological approaches could significantly improve large-scale production and enhance the tunability of BC properties. Additionally, these approaches may simultaneously provide environmental benefits, depending on their future progresses. Future advancements should prioritize circular fermentation and biotechnological techniques to expand the potential of BC for sustainable industrial applications.

## 1. Introduction

For decades, synthetic polymers have dominated applications in food packaging, textiles, biomedical, engineering, and agriculture [1,2], revolutionizing multiple technical, industrial, and social sectors. However, their non-renewable nature, slow biodegradability, and challenging waste management remain major drawbacks [3]. Consequently, plastic pollution continues to harm ecosystems, wildlife, and human beings, thus further exacerbating the global plastic crisis [4].

Notably, the textile sector is one of the largest consumers of synthetic polymers. Synthetic textiles and leathers are primarily made from synthetic polymers such as polyvinyl chloride (PVC) and polyurethanes (PU) [5]. These materials contribute significantly to environmental stress by releasing microplastics during use and disposal [3]. Given this impact, there is a clear need for the textile sector to alleviate the global environmental pollution crisis [6,7]. This can be achieved by replacing synthetic polymers with green, renewable, and sustainable biopolymers suitable for industrial applications and scalable for widespread use [8,9,10,11,12,13].

Together with the environmental stress caused by the extensive use of plastics, the total volume of food waste is estimated at 1.3 to 1.4 billion tons, with projections indicating it could rise to 2.6 billion tons by the end of 2025 [13]. Therefore, food waste should be valorized in a circular economy scenario, integrating sustainable technologies with the zero-waste principle [14]. Research is striving to employ agro-industrial waste biomass in biochemical processes to produce bio-based polymers, addressing the urgent need for plastic alternatives and the growing issue of food waste. These processes exploit microbial fermentation to produce biopolymers, offering a sustainable alternative to synthetic materials [15,16,17].

Among the different microbial-derived biopolymers, bacterial cellulose (BC) has gained considerable attention in both research and industry as a promising contributor to the bioeconomy as an alternative to synthetic polymers and plant-derived cellulose [1]. Moreover, since agri-food waste can serve as a source of nutrients for bacteria fermentation [9,18], BC offers a viable solution for the circularity of food industry waste. However, since these processes are still experimental, BC industrial applications and scalability have yet to be fully defined and optimized.

Laying upon these challenges, the present work is based on the urgent need for valuable alternatives to synthetic polymers, to upcycle agro-industrial waste, and to scale-up and control BC production for industrial applications.

Significant progress has been made in BC research over the past few years, from developing production strategies and cost-effective media formulations [1,19] to exploring increasingly complex applications and sustainability assessments. Recent works have focused on specific aspects of BC, such as its industrial applications [20] and waste-based media as cost-reduction strategies [9,18].

**Figure 1:**
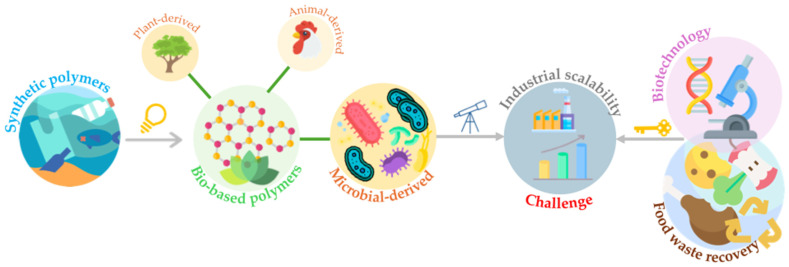
The study’s rationale is based on the problem of food and plastic waste. The biotechnology–circularity–scalability nexus must be considered to allow the industrial scalability of microbial-derived polymers.

Our focus is on integrating circular and biotechnological approaches to enhance large-scale BC production, improve material properties’ tunability, and simultaneously deliver environmental benefits. Upcycling food waste for microbial fermentation fits within a circular economy framework, boosting BC production. Additionally, biotechnology techniques have been reported as key factors in enhancing BC yield and tailoring the physico-chemical properties of BC [9]. Therefore, the biotechnology–scalability–circularity nexus is crucial for BC’s large-scale industrial application (Figure 1).

Finally, a case study of the fast-fashion phenomenon is proposed, demonstrating how the emerging BC-based materials trends could mitigate the clothing industry’s environmental concerns from a circular and sustainable economy perspective.

## 2. Bacterial-Derived Materials

Biopolymer synthesis primarily relies on microorganisms, such as bacteria and yeasts, due to their ease of cultivation, genetic manipulability, and ability to produce a wide range of materials [21]. Bio-gels are key components of the extracellular polymeric matrix (EPM) in microorganisms like *Achromobacter*, *Pseudomonas*, *Komagataeibacter*, *Agrobacterium*, *Salmonella*, and *Aerobacter* [22]. Microbial-derived bio-gels gained significant research and industrial attention due to their different viscosities, rheological properties, and ability to swell and interact with specific structures, enabling various applications in biomedicine, electronics, and fashion [4,23]. Bacterial-derived bio-gels can be tailored to expand their application possibilities [23]. Synthetic biology integrates engineering principles with biology to design and construct new biological systems and organisms, developing sustainable methods for producing tailored bio-gels. Notable examples are hyaluronic acid, xanthan gum, and bacterial cellulose.

*Hyaluronic acid* (*HA*) has various applications across various industries, driving a rapidly growing market demand that shows a steady upward trend [24]. Initially, HA was extracted from the umbilical cord, skin, and synovial fluid [24,25]. However, over time, microbial production of HA has become the dominant approach [26]. Notably, the primary microorganism used in HA production is *Streptococcus zooepidemicus* [27,28].

With advancements in the understanding of microbial polysaccharide biosynthetic pathways and associated genes, genetically engineered bacteria have been developed to enable low-cost HA production [24,27,29]. Alternative substrates derived from agricultural byproducts [30], such as sugar beet pulp and wheat bran, and waste products from industrial processes [31], like distillery waste and molasses, are being considered. These alternatives are potentially more cost-effective than traditional substrates, and they may represent interesting alternatives for commercial applications [27].

*Xanthan gum* is a heteropolysaccharide produced by the bacterium *Xanthomonas campestris* [4,32]. Traditionally, xanthan gum has been derived from bacterial fermentation, but the high manufacturing cost poses significant challenges to its microbial production. To address this, alternative low-cost substrates, such as food industry and agricultural wastes, have been explored to reduce production expenses [33,34]. Substrates like citrus waste, carob extract, olive mill wastewater, waste sugar beet pulp, corn steep liquor, apple juice residue, vegetable scraps, and whey permeate have been employed for xanthan gum biosynthesis [35].

BC is a promising contributor to the bioeconomy, offering an alternative to synthetic polymers and plant-derived cellulose [1]. Like HA and xanthan gums, the main challenge with BC is implementing efficient and cost-effective industrial methods for microbial cellulose production [19,20]. From an economic and sustainable perspective, BC provides a viable solution for the circularity of agro-industrial waste, as these wastes can serve as nutrient sources for microbial fermentation [9,18]. However, since these processes are still experimental, BC production’s industrial applications and scalability have yet to be fully optimized.

## 3. Bacterial Cellulose: Features and Biosynthetic Pathway

**Figure 2:**
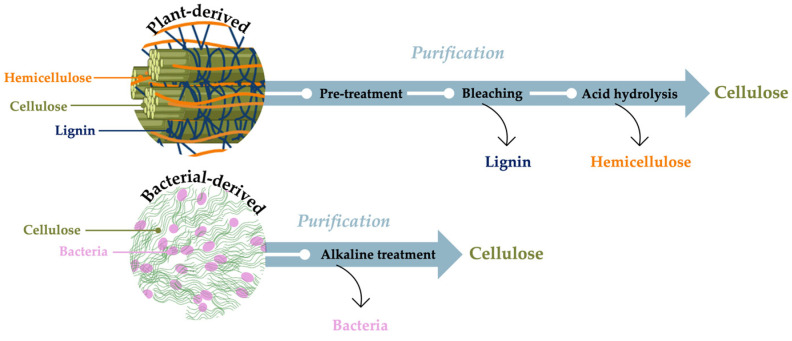
Plant-derived cellulose is linked to lignin and hemicellulose, which require strong chemical processes [61]. Bacterial cellulose (BC), produced in a purer form, requires a straightforward purification process. Reprinted with permission from [62] under the CC-BY 4.0 publishing license terms.

Cellulose is the most abundant biopolymer on Earth as a key component of plant cell walls [3,36,37]. Cellulose can be obtained through two primary processes: chemical and biochemical [38]. The chemical process extracts cellulose from lignocellulosic biomass containing hemicelluloses, lignin, pectin, and other biogenic contaminants. Since these unwanted compounds are tightly bound to the cellulose, their removal requires complex and costly chemical treatments [39] (Figure 2). These treatments include acid or alkaline pretreatments and bleaching, contributing to environmental pollution due to high energy consumption and hazardous chemicals [9,15,38,40,41].

In addition to plants, cellulose can be produced through biochemical processes by microorganisms that thrive in biofilms. Biofilms are microbial communities embedded within a self-produced extracellular polymeric matrix (EPM). Some microorganisms synthesize cellulose as EPM, providing structural support and cell protection [42]. In these cultures, cellulose forms an extracellular gel-like sheet at the air–liquid interface.

Several bacteria are known to produce cellulose, primarily as a structural component of their biofilms. The most well-studied cellulose-producing bacteria include the gram-positive bacterium *Sarcina ventriculi* and gram-negative bacteria, such as *Achromobacter*, *Pseudomonas*, *Komagataeibacter*, *Agrobacterium*, *Salmonella*, *Aerobacter*, *Azotobacter*, *Alcaligenes*, and *Rhizobium*. Among them, the aerobe and acetic acid bacterium *Komagataeibacter hansenii* (formerly *Gluconobacter xylinus*) is the most efficient cellulose producer and the model organism for BC research and applications [22]. A single bacterium can polymerize 200,000 glucose molecules into β-1,4-glucan chains per second while assembling these polymeric chains into nanofibers [43]. *K. xylinus* has been observed to produce BC in various sugar- and ethanol-rich environments, including fruits, vinegar, nata de coco, and kombucha tea [44]. In these niches, the bacterium forms cellulose as part of the EPM on the surface of fermenting liquids.

BC is synthesized by a membrane protein complex called cellulose synthase through three key steps: (1) the polymerization of glucose units via *glycosidic linkages* to form β-(1,4)-glucan chains, (2) the extracellular transport and assembly of these β-(1,4)-glucan chains into the growth medium, and (3) the crystallization of cellulose fibrils, where intra- and intermolecular hydrogen bonds form between the chains, leading to the organization of the fibrils into highly ordered structures [45].

Although the BC synthase (*bcs*) operon shows variable genetic organizations across different species [46], in many cellulose-producing acetic acid bacteria, including those belonging to the genus *Komagataeibacter*, the *bcs* operon consists of four subunits: BcsA, BcsB, BcsC, and BcsD. BC synthesis begins at the periplasmic membrane, where the catalytic unit formed by BcsA and BcsB proteins facilitates the condensation of activated glucose monomers with the reducing end of the nascent glucan chain [47,48]. This process is regulated by the allosteric activation of BcsA through cyclic di-GMP, a universal bacterial second messenger involved in biofilm formation.

The glucan chain is then translocated through the periplasmic membrane in the outer membrane via BcsC [45]. In the periplasm, the BcsD protein assembles the cellulose molecules into crystalline fibers by forming an octamer capable of binding up to four glycan chains. Disruption of the BcsD-coding gene in mutant strains impairs the quantity and crystallinity of the secreted cellulose [49]. Accessory genes located in the flanking regions of the *bcs* operon, which are not fundamental to BC biosynthesis, play a crucial role in the proper formation of glucan chains. For example, the *cmcAx* gene encodes an endo-β-1,4-glucanase that selectively degrades amorphous and disordered cellulose while remaining ineffective against crystalline cellulose. This enzyme promotes proper crystallization during cellulose synthesis by breaking down tangled structures, ultimately improving overall cellulose production [50].

BC’s primary structure consists of long-chain (1→4) linked β-D-glucan chains regardless of its natural source. These chains grow from the nanoscale to the macroscopic scale, forming a 3D network with a polymerization degree of up to 20,000 [51]. The chain linkages create an extended secondary structure with a unique ribbon-like shape, while the BC tertiary structure is stabilized by intermolecular hydrogen bonds and van der Waals forces [52].

BC synthesis is more efficient than plant-derived cellulose in terms of extraction and purification. Unlike plant cellulose, BC is produced without impurities such as lignin and other compounds [3,15,38,53,54,55]. This simplifies the purification and scaling-up processes, eliminating the need for extensive mechanical, enzymatic, or chemical treatments to remove contaminants and achieve nanoscale dimensions [40]. While chemically similar to plant cellulose, BC offers distinct advantages, including a greater degree of polymerization and water-holding capacity, an ultrafine interwoven structure, high tensile strength, elasticity, crystallinity, and biocompatibility (Figure 2) [8,15,38,40,53,56,57]. In addition, the quality and yield of plant-derived cellulose depend heavily on species and growing conditions, whereas BC permits more controlled and tunable production conditions [58,59]. Finally, BC is an inexhaustible, renewable, and environmentally friendly material [40].

BC’s chemical structure allows for various functionalization and chemical modifications. These features make BC an ideal candidate for developing bio-based materials to address global challenges [3,9,40]. Additionally, recent engineering advancements have improved the cost-effectiveness and accessibility of BC, positioning it as a practical alternative to traditional plastics [60].

## 4. Towards Industrial Production of Bacterial Cellulose

Ongoing research and optimization of BC’s production processes are essential for expanding its applications and enhancing its competitiveness against synthetic materials [3,40,63]. A bibliometric analysis was conducted to highlight BC’s research trends and advancements.

**Figure 3:**
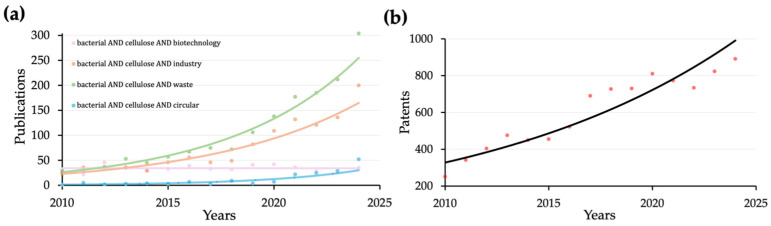
(**a**) Bibliometric analysis of different topics related to BC. (**b**) Patent about BC publications analysis from 2010 to 2024. The Scopus (Elsevier, NL) database was selected as the source of peer-reviewed literature. The search strategy involved the queries: “bacterial AND cellulose AND biotechnology”, “bacterial AND cellulose AND waste”, “bacterial AND cellulose AND circular”, “bacterial AND cellulose AND industry”.

The bibliometric analysis revealed a linear increase in research activity related to BC in biotechnology. Notably, since 2010, there has been an exponential rise in studies focused on BC’s industrial applications and waste recovery, indicating a clear shift towards optimizing its large-scale production and sustainable utilization. Furthermore, research on BC in the context of circularity has accelerated since 2020, underscoring a growing commitment to integrating BC into sustainable industrial frameworks (Figure 3a). These findings suggest a concerted effort within the scientific community to push the boundaries of BC production [64], aiming to enhance its feasibility for commercial applications and foster innovation in biotechnology.

To analyze the progression of BC-related patent publications, patents from 2010 to 2024 were retrieved from the Espacenet database using targeted keyword searches (“bacterial cellulose”). The search results displayed a consolidated exponential increase in BC-related patents (Figure 3b), indicating a growing interest in the technological transfer from laboratory research to industrial-scale applications [36].

The combined bibliometric and patent analysis confirms a notable shift in BC research and innovation, marking its transition from fundamental studies to large-scale industrial applications. The surge in publications, particularly after 2020, aligns with increasing interest in sustainability and circular economy frameworks. These trends underscore a broader scientific and market-driven effort to expand BC’s feasibility for commercial applications, fostering innovation in biomaterials, waste recovery, and sustainable manufacturing.

### 4.1. Biotechnological Process to Boost Bacterial Cellulose Production Yield

**Figure 4:**
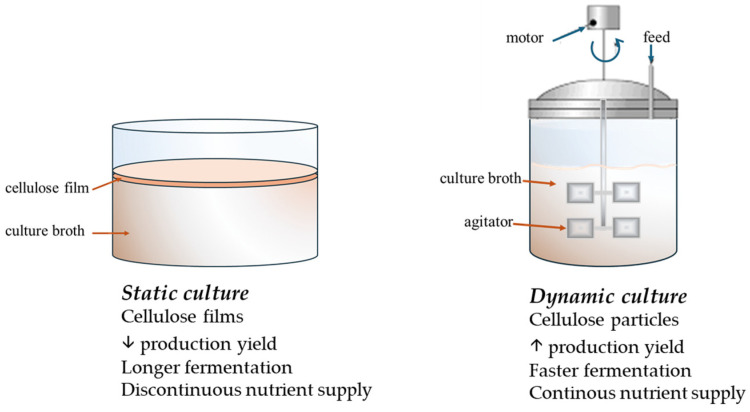
Features of static and dynamic cultivation methods of bacterial cellulose.

BC cultivation can generally be carried out using static or agitated methods (Figure 4). Cellulose films biosynthesized in static cultivation have a film-like formation that requires long incubation periods yet low yields [65]. In contrast, cellulose produced by dynamic fermentation comprises particles with high yields but limited applications [3,66].

Recent biotechnological advances have increased BC yield and quality to meet the growing global demand for BC-based products. Over the years, conditions for BC production have been economically optimized, such as new strain isolation [67,68,69], media composition optimization [70,71,72], cultural conditions refinement [73], and operational methods and bioreactor design improvement [74]. Additionally, BC production can be improved by co-culturing different microorganisms, where the non-BC-producing microorganisms create conditions to boost BC synthesis or improve the mechanical properties of BC nanocomposites. For example, co-culturing *Bacillus cereus* and *K. xylinus* in corn stover enzymatic hydrolysate led to a fourfold increase in BC production. The findings indicated that acetoin and 2,3-butanediol, produced by *B. cereus*, were key factors in increasing BC yield [75]. Similarly, co-cultivating cellulose-synthesizing bacteria with lactic acid bacteria significantly boosted BC production, resulting in a twofold increase in dry weight compared to the monoculture of cellulose-synthesizing bacteria [76]. Notably, the cellulose produced through co-cultivation exhibited superior quality to that obtained from the monoculture. In another experiment, BC with a threefold increase in modulus was obtained by co-culturing *K. hansenii* with the pullulan-producing *Aureobasidium pullulans* [77]. Pullulan, a polysaccharide containing α(1–4) and α(1–6) glycosidic bonds, can bind with BC microfibrils, thereby improving the mechanical properties of BC nanocomposites.

However, co-culturing bacterial strains to produce BC for industrial applications presents some aspects that need future refinement. For instance, a deeper understanding of how co-cultured microorganisms respond to various nutrient combinations is essential for enabling large-scale production. Additionally, further studies on the interactions and competition between inoculated microorganisms are necessary to develop scalable processes of BC fermentation. Finally, future optimization efforts could focus on factors such as salt concentrations, bioreactor designs, and the extent of contact between the inoculated microorganisms [77].

Synthetic biology tools and metabolic engineering improve large-scale BC applications. With the advances in genome sequencing and synthetic biology toolkits [78,79,80], some BC-producing bacteria have been successfully engineered. These engineered bacteria have not only augmented BC yield and productivity [81,82,83] but also bettered cellulose structural properties [84] and enabled in vivo functionalization [85]. Various genetic engineering strategies have been employed to enhance BC production in *K. xylinus*. These include designing BC producers with synthetic ribosome binding sites capable of tunable gene expression [86], modifying the BC biosynthesis pathway and related pathways [87], overexpressing genes in the cellulose synthase operon [88], and enhancing oxygen tolerance by overexpressing the aerobic respiration control protein A [89]. The recombinant expression of mannose kinase and phosphomannose isomerase genes from *E. coli* has enabled *K. xylinus* to utilize mannose for BC production, providing a strategy to boost BC yield. Recombinant bacteria were engineered to efficiently metabolize mannose-rich biomass, providing a cost-effective alternative to glucose [90]. This approach involved modifying the mannose catabolic pathway in *K. xylinus* ATCC 23770 by introducing mannose kinase and phosphomannose isomerase genes from *Escherichia coli* K-12. When mannose was used as the sole carbon source, the recombinant strain exhibited an 84% increase in BC yield [90]. Additionally, BC tensile strength and elongation improved 1.7-fold, while Young’s modulus increased 1.3-fold compared to the wild-type strain. Further improvements in BC production were achieved by overexpressing the *bcsC* and *bcsD* genes in *K. xylinus* ATCC 700178 and modifying the culture method to reduce medium viscosity by adding water during fermentation [88]. The *bcsC*-overexpressing strain produced 5.2 g/L of BC, doubling the wild-type yield of 2.2 g/L while maintaining BC properties. Overexpressing *bcsD* improved BC crystallinity, degree of polymerization, mechanical strength, and thermal stability. When both genes were overexpressed, the BC yield reached 6.8 g/L, a threefold increase over the wild-type yield.

### 4.2. Biotechnological Approaches to Tailor Bacterial Cellulose Properties

Cellulose-producing bacteria can be genetically engineered to tailor BC’s chemical and mechanical features. For instance, overexpressing the motility genes *motA* and *motB* in *K. hansenii* resulted in a more flexible BC fiber structure, making it well-suited for tissue engineering applications [91]. In contrast, knocking out *motA* and *motB* genes in *K. hansenii* produced denser, more compact nanocellulose fibers with superior mechanical properties compared to the wild-type strain [92]. These findings suggest that modifying motility-related genes indirectly influences cellulose nanostructure. CRISPR technology has been applied to *K. xylinus* to modulate the expression of the *galU* gene, which encodes UGPase—a key enzyme that regulates carbon flux between BC synthesis and the pentose phosphate pathway [93]. By adjusting *galU* expression levels, researchers could control BC’s structural properties, including porosity and crystallinity. A BC/HA copolymer was obtained with *K. hansenii* by introducing the hyaluronan synthase gene from *Pasteurella multocida* ATCC 15742 and the UDP-glucose dehydrogenase gene from *Sinorhizobium meliloti* 1021 [94]. During the production of the BC/HA hybrid, BC was the main component forming the matrix, with HA incorporated into the matrix structure. Another approach for producing BC/hyaluronic acid composites involved co-culturing *K. hansenii* with an engineered *Lactococcus lactis* strain. This strain was modified to express the heterologous *has*ABC genes from *Streptococcus zooepidemicus*, which encode key enzymes in the hyaluronic acid synthetic pathway [95]. By expressing the curdlan synthase gene from *Agrobacterium* sp. ATCC 31749 in *K. xylinus*, researchers successfully produced a BC/curdlan composite with reduced water permeability [96]. While curdlan modified BC’s surface morphology and porosity, it did not affect the fiber crystallinity. This suggests that cellulose synthesis and excretion remained dominant, even as the engineered strain produced and secreted both polymers. Furthermore, co-cultured *Acetobacter oryzoeni* MGC-N8819 with the efficient nicotine-degrading mutant strain *Pseudomonas* sp. JY-Q/5 Delta boosted BC production from tobacco waste extract [97].

In vivo functionalization of BC allows surface modifications to enhance cellulose functionality. A synthetic symbiotic co-culture of bacteria and yeast was developed by co-cultivating *K. rhaeticus* with *Saccharomyces cerevisiae* to functionalize BC pellicles using engineered yeast strains [98]. In this system, sucrose served as the carbon source, with *K. rhaeticus* using the glucose released through sucrose hydrolysis by yeast-secreted invertase. To achieve functionalization, three engineered yeast strains were used, each secreting a specific enzyme: β-lactamase (BLA), α-galactosidase (Mel1), and laccase from *Coriolopsis trogii* (CtLcc1). These enzymes were fused with a cellulose-binding module from *Cellulomonas fimi* to enhance retention within the BC pellicles, thereby reducing enzyme leakage and increasing enzymatic activity. The success of functionalization was demonstrated through distinct color changes in the BC-based gels: yellow upon the addition of the BLA substrate nitrocefin, blue from X-α-Gal (5-bromo-4-chloro-3-indolyl-β-d-galactopyranoside) digestion by Mel1, and dark green due to ABTS (2,2-azino-bis-3-ethylbenzothiazoline-6-sulfonic acid) oxidation by CtLcc1.

Another strategy for in vivo BC functionalization involves introducing genetically engineered expression vectors into the host, enabling the fusion of a target protein with one of the four cellulose-binding modules (CBMs): CBDCex, CBDclos, CBDcipA, and dCBD. These CBMs are short peptides that firmly bind to cellulose fibrils, enhancing protein attachment to the cellulose matrix [99]. For example, a chimeric protein combining a CBM and an adhesion peptide was engineered to improve human microvascular endothelial cells (HMEC) binding to BC. In this modified BC-HMEC system, HMEC cell growth was inhibited, and the HMEC cells adopted an elongated morphology, forming cord-like structures [85].

## 5. Cost-Effective Production of Bacterial Cellulose

BC production requires a nutrient-rich fermentation medium that provides a well-balanced supply of carbon sources (like glucose, fructose, and sucrose), nitrogen sources (like peptone), essential minerals, and vitamins—especially B vitamins. These nutrients support bacterial growth and enhance the yield and quality of the produced cellulose [100,101]. The traditional production process is expensive, with the culture medium alone accounting for approximately 30% of the total production cost [38,40,102]. Conventional BC production relies on costly synthetic media, such as the Hestrin–Schramm (HS) medium. To reduce costs, researchers are exploring low-cost substrates [13], particularly in the cases of kombucha-derived and nata de coco-derived BC [103]. Below are reported low-cost BC production methods that leverage bacterial fermentation for food and beverage production.

### 5.1. Nata De Coco

Nata de coco is a traditional fermented food with hydrogel-like features. It consists of BC produced at the liquid–air interface through the static fermentation of coconut water [104]. The acetic acid bacterium *Komagataeibacter nataicola*, abundant in traditional nata de coco, is widely recognized as the key microorganism responsible for *BC* production.

Recent studies have explored the biological factors influencing *K. nataicola* strains in BC production by analyzing pre-fermented coconut water’s microbial community structure and metabolite profile [105]. The findings revealed that the lactic acid bacteria *Limosilactobacillus*, *Lactiplantibacillus*, and the yeasts *Saccharomyces* and *Candida* enhance BC yield by *K. nataicola*. Additionally, ethanol, lactate, and mannitol were found to promote BC synthesis, whereas ascorbate inhibited BC production or had no significant effect, depending on *K. nataicola* strains.

Given the wide range of applications for this type of cellulose, different strategies have been investigated to improve BC productivity. For instance, proteomic analysis was performed to assess the metabolic processes involved in BC production during nata de coco fermentation, revealing that ethanol supplementation increased yield by 48% [106]. Genome analysis was conducted to investigate the genetic sequences of bacteria isolated from nata de coco. Additionally, the study highlighted variations in cellulose production and explained the instability of genetic traits observed in *Komagataeibacter* and related acetic acid bacteria.

However, the role of coconut water characteristics in the biological properties of bacterial strains and the structure of BC remains unclear, leading to inconsistencies in BC production and quality [105,107]. Specifically, the use of nata de coco is predominantly limited to food applications, and research exploring its potential uses remains undeveloped [108].

### 5.2. Kombucha Tea Fermentation

**Figure 5:**
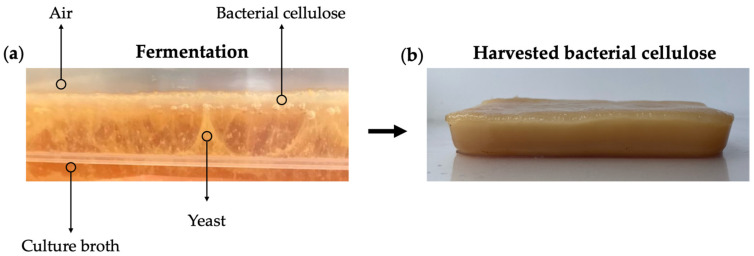
(**a**) A symbiotic culture of bacteria and yeast during kombucha tea fermentation (**b**) BC at the end of the fermentation process. Original images of BC produced by the authors through kombucha tea fermentation.

The global kombucha market has grown in recent years, reaching 1.84 billion USD in 2019, and it is expected to expand by 23.2% by 2027 [109]. BC is a natural byproduct of kombucha tea fermentation, a process in which a Symbiotic Culture of Bacteria and Yeast (SCOBY) ferments sweetened black or green tea for 10 to 14 days, resulting in a mildly alcoholic, probiotic-rich beverage. As a result, kombucha fermentation is emerging as a promising source of BC. During kombucha fermentation, a thick, floating cellulose-based biofilm develops at the air–medium interface [13,110,111] (Figure 5a). This cellulose-based EPM serves as a protective barrier, shielding the microorganisms from external contamination and maintaining microbial balance within the culture.

**Figure 6:**
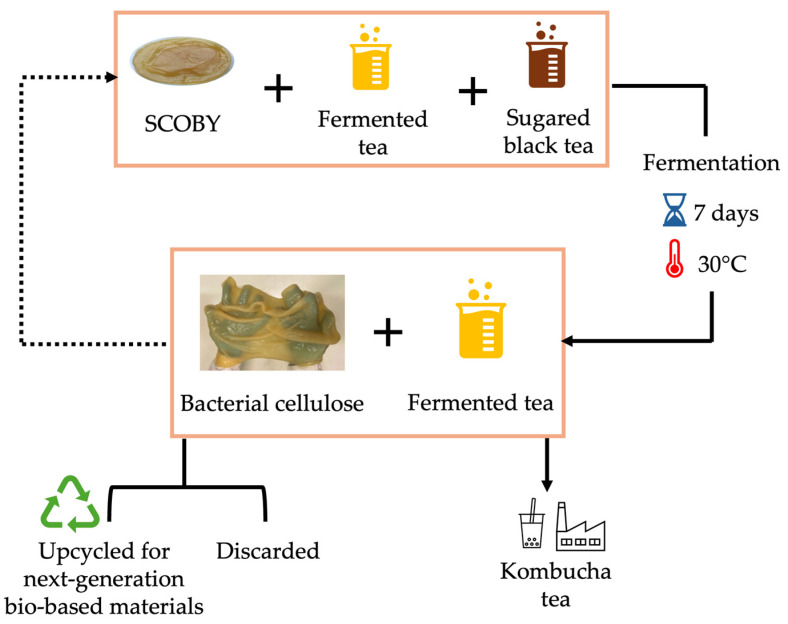
Schematic process of BC production through kombucha tea fermentation. Sugared tea, fermented tea (kombucha tea), and SCOBY (formerly BC) are mixed and incubated for at least 7 days under controlled temperature. The solid and dotted arrows underline the circularity of BC production through kombucha tea fermentation, where a portion of the bio-synthesized SCOBY is used as inoculum in subsequent fermentations. Additionally, the remaining SCOBY can be upcycled into bio-based materials. Original images of BC were produced by the authors through kombucha tea fermentation.

While some of the BC produced (Figure 5b) is employed as an inoculum for subsequent fermentations, it is often discarded and considered an industrial byproduct of kombucha tea production (Figure 6) [112,113,114]. The potential of kombucha-derived BC in the circular economy is further strengthened by using tea waste as a nitrogen source, enabling a zero-waste culture medium [115].

Traditionally, ethanol, organic acids, and additional carbon and nitrogen sources are added to the fermentation medium to enhance BC production. Furthermore, acetic acid bacteria cannot transport large molecules like disaccharides and oligosaccharides across the cell membrane, so these biopolymers must be hydrolyzed before being used [116]. However, during kombucha fermentation, these conditions are naturally met by the microbial consortium without incurring additional costs. The symbiotic microbiota of SCOBY is composed of acetic acid bacteria, primarily *K. xylinus*, lactic acid bacteria (*Lactobacillus*, *Lactococcus*), and several yeast species, such as *Zygosaccharomyces*, *Torulaspora*, *Brettanomyces*, *Zygosaccharomyces*, and *Pichia* [117].

Yeasts produce the enzyme invertase, which breaks down sucrose into glucose and fructose. The yeasts then ferment glucose into ethanol and carbon dioxide. Lactic acid bacteria generate lactic acid and induce α-galactosidase to break down oligosaccharides [118]. The resulting monosaccharides and ethanol serve as a substrate for acetic acid bacteria, which form cellulose and produce organic acids such as acetic, gluconic, and glucuronic acids. As monosaccharides in the medium become depleted, the population of invertase-producing yeast increases. Simultaneously, ethanol produced by yeast stimulates the BC synthase enzyme, promoting the formation of the cellulose film in a virtuous cycle. Studies suggest that ethanol is an energy source for ATP production, reducing the need for glucose in energy generation. This allows cells to utilize glucose more efficiently for cellulose synthesis [119]. Additionally, ethanol and acetic acid in the medium act as antimicrobial agents that prevent the growth of pathogenic microbes and molds [120]. Tea compounds like caffeine further stimulate BC production by activating cellulogenic bacteria complexes. Moreover, vitamins and other nutrients released through yeast autolysis enhance bacterial activity, further supporting BC synthesis.

Unlike yeast, acetic acid bacteria require large amounts of oxygen for growth and activity. Bacteria consume dissolved oxygen in the liquid phase to form cellulose, which rises to the air–liquid interface. At this interface, acetic acid bacteria utilize atmospheric oxygen to produce new cellulose layers atop the existing ones, generating a floating 3D BC. As fermentation progresses, the biofilm thickens while remaining suspended in the fermentation broth as long as oxygen is available. Once oxygen becomes limited, BC production ceases, and the bacteria enter a quiescent state in both the biofilm and liquid broth. These dormant bacteria can be reactivated by adding sweetened tea extract or used as inoculum for subsequent fermentation batches (Figure 6).

Several factors are crucial in optimizing BC production during kombucha fermentation. These include the composition of the kombucha consortium [54], the type of carbon and nitrogen sources [54,121,122,123], pH levels, dissolved oxygen, fermentation duration, temperature [111], and the geometry of the fermentation vessel [35,124,125,126]. For instance, studies have assessed the effects of different nitrogen sources (black, white, and green tea) and carbon sources (sugar cane, palm, brown sugar, and honey) on kombucha-derived BC production and properties [127]. The results showed that green tea and palm sugar produced the thickest BC (0.2 mm) with the highest tensile strength (24 MPa). Recently, SCOBY production was optimized using cost-effective substrates through the Taguchi method and response surface methodology [128]. Replacing tea leaves with tea dust as a nitrogen source and using blackstrap molasses as the carbon source significantly optimized kombucha production while reducing cost per liter from USD 0.075 to USD 0.027. The optimal conditions for maximum BC yield were 75 g/L molasses, 10 g/L tea, 20 g/L SCOBY inoculum, pH 5, and an incubation temperature of 30 °C. Under these conditions, the SCOBY growth rate increased slightly (1.142 kg/day vs. 1.136 kg/day in the control), while production costs were reduced by an impressive 97.8% [128].

In 2024, a three-factor Box–Behnken design was employed to identify the optimal levels of three key variables—sucrose concentrations, kombucha inoculum percentage, and fermentation temperature—for BC production from kombucha-fermented soy whey. The results revealed the optimal fermentation conditions as 8.5% sucrose, 10% kombucha inoculum, and a fermentation temperature of 32 °C. Under these conditions, the BC yield reached 4.20 g/100 mL (dry weight) after 11 days of fermentation [129]. Furthermore, the resulting BC demonstrated excellent tensile strength and a high water absorption capacity.

The impact of inoculum age and concentration on BC yield in kombucha-type fermentation was investigated using green acerola residue extract. Lower inoculum concentrations reduced BC yield, while higher concentrations increased production by 3.2 times. A 3-day-old inoculum produced the lowest BC, whereas older inoculums (up to 12 days) showed decreased metabolic activity. The optimal conditions were identified as a 6-day-old inoculum at 7.5% concentration, which yielded up to 2.2 g/L of BC with 70 g/L of carbon source [130].

Currently, no standardized protocol exists for fermenting kombucha tea to produce BC. Reported daily yields are approximately 0.5 g/L×day [131]. However, adjusting the fermentation variables can significantly improve BC production efficiency and increase daily yields.

However, the limited information on process feasibility often hinders the large-scale production of kombucha-derived BC. A techno-economic analysis of a kombucha-based cellulose production facility with an annual capacity of 60 tons was evaluated. The economic analysis estimates a total investment of USD 13.72 million and operating costs of USD 3.8 million, with 89% of expenses attributed to facility and labor costs. The process feasibility is demonstrated by a payback period of 4.23 years, a 23.64% return on investment, and a 16.48% internal rate of return [103]. Sensitivity analysis indicates that increasing fermentation unit volumes and automating the process could significantly reduce input costs.

Scalability remains a major challenge in BC production from kombucha. While many studies emphasize the potential for scaling up BC biosynthesis and production, no proof of concept has yet been demonstrated at an industrial scale. Additionally, additives that enhance BC yield during fermentation often render the resulting kombucha beverage undrinkable. Therefore, the ideal solution would be to optimize and standardize a protocol that maximizes BC production while maintaining the fermented beverage’s drinkability.

## 6. Circular Production of Bacterial Cellulose

Numerous studies have explored using organic wastes and byproducts as alternative feedstocks to reduce BC production costs and integrate BC production into the circular economy. These byproducts, generated at various stages of agro-industrial processes, serve as valuable reservoirs of key components for cellulose biosynthesis [9,15,38,132,133]. Using waste biomass rich in carbon and nitrogen supports BC production and reduces environmental pollution, enabling cost-effective, resilient, and sustainable bioprocesses [3,15,134]. Table 1 summarizes studies that have explored agro-industrial waste and byproducts as a nutrient source for bacterial fermentation. Reported BC production yields vary significantly, primarily depending on fermentation container dimensions, particularly the surface area/depth ratio [35].

For example, the effectiveness of banana pulp in a 1:2 ratio in the culture broth was demonstrated, yielding 30 g/L of BC [135]. Other fruit-derived substrates have also been tested, including pineapple waste [136], which achieves a BC production yield of 3.24 g/L, outperforming the traditional HS medium (1.98 g/L). Diverse fruit juices, such as orange, grape, apple, pineapple, and pear, were assessed as additives in fermentation media, with orange juice showing the highest sugar-to-cellulose conversion efficiency [137]. In a similar approach, cheese whey and olive mill waste were employed as circular feedstocks for BC production. While olive mill-derived BC exhibited finer fibers and a well-structured cellulose network, cheese whey resulted in the highest production efficiency [138]. Additionally, the abundance of bread waste led to optimizing a cost-effective fermentation medium suitable for BC production and large-scale industrial applications. The biosynthesized BC displayed high crystallinity, fine fibers (40 nm), excellent thermal stability, and water-holding capacity [139]. BC-producing bacteria have also been isolated from fruits and vegetables such as strawberries, tomatoes, apples, pomegranates, pineapples, and orange waste. Moreover, hydrolyzed fruit peels, including apple, citrus, banana, mango, and pomegranate, were used to produce a circular fermentation medium, with the HS medium as a control. Strawberry-derived bacteria displayed the highest BC yield (0.48 g/L), and the zero-waste medium produced BC with greater crystallinity than that obtained with the traditional HS medium [140].

Among the diverse agro-industrial byproducts, residues from the wine and pulp industry proved to be the most effective sources for BC production, yielding 0.6 g/L and 0.3 g/L, respectively, followed by biodiesel crude residue and cheese whey [141]. Similarly, BC production was optimized using carob and haricot beans, achieving yields of 1.8 g/L with ideal properties and 3.2 g/L with non-optimal mechanical properties, though still suitable for use [142]. Beet molasses, vinasse, and waste beer fermentation broth were adopted as circular fermentation media for BC production as an alternative to the HS medium. The findings revealed that beet molasses and vinasse reduced polymer crystallinity while increasing the degree of polymerization, whereas waste beer fermentation broth had the opposite effect [143].

Several studies have explored unconventional circular carbon sources with high BC conversion efficiencies. For example, coffee cherry husk was utilized as a nutrient source to achieve a BC production yield of 8.2 g/L, surpassing synthetic media [144]. Similarly, fig waste was adopted as a substrate for BC production, yielding 8.45 g/L under optimized conditions, one of the highest production yields reported to date [15]. Finally, maple syrup has also been identified as a viable carbon source, with a BC production yield of 1.51 g/L, comparable to pure fructose (1.60 g/L) [145]. Pineapple peel juice led to a BC production yield of 2.8 g/L, slightly outperforming the HS medium (2.1 g/L) [146]. Pineapple peels and banana extract served as alternative nutrient sources for BC-producing microorganisms, supporting BC production of up to 2 g/L). The BC production yield with uncommon substrates was higher than the 0.5 g/L achieved with the HS medium (0.5 g/L) [147]. Hydrolyzed vegetable waste, such as asparagus, was also employed as a fermentable sugar source in the culture medium. Several inoculum doses and incubation times were tested, leading to an optimized protocol that achieved a BC production yield of 2.57 g/L [148]. Among different organic waste sources—including tea, vegetables, and fruit peels—fruit wastes yielded the highest BC production of 25 g/L [115].

A glucose-rich solution was prepared by acidic hydrolysis of stale bread, while a fructose- and glucose-rich extract was obtained from waste apple pulp. The apple pulp was employed to cultivate *K. xylinus* as a sole feedstock or supplemented with the HS medium. The supplementation significantly optimized BC production, yielding 3.4 g/L with the apple pulp extract and 2 g/L with the stale bread hydrolysate [149].

These findings highlight the primary challenges related to biotechnology, particularly the need to improve productivity to meet industrial demands. While ongoing research continues to optimize BC yield for large-scale production, most reported studies remain at the experimental stages of these processes, and industrial applications are not yet fully defined [59]. Food waste is a promising opportunity as a sustainable and circular nutrient source for BC production. Although organic wastes have been shown to yield higher BC production than traditional culture media, further research is needed to optimize fermentation processes for industrial scalability.

**Table 1:** Studies employing agri-food waste as a nutrient source for bacterial fermentation. The reported production yields are calculated on the dry BC weight. The yield increase was calculated based on the yield employed in the traditional HS fermentation medium. (**) not specified if dry or wet yield.

Agri-Food Waste and Byproducts as Substrate	Application	Dry Production Yield	Yield Increase	Industrial Production	References
Wastewater from rice wine	-	1.83 g/L	+26%	-	[150]
Potato peel	-	4.7 g/L	+288%	Favorable results using potato peel waste for cost-effective industrial production	[151]
Banana leaves	Cardboard paper	30 g/L (**)	-	Tunable properties for industrial needs	[135]
Coffee husk	-	8.2 g/L	+446%	-	[144]
Wheat straw	-	-		-	[152]
Pineapple	-	3.24 g/L	+63%	-	[136]
Fruit juices	-	Up to 6 g/L	-	-	[137]
Rotten fruits and milk whey	Different envisioned applications	60 mg/mL	+100%	-	[141]
Grape, cheese whey, and sulfite pulping liquor	-	Up to 2.5 g/L	-	Further characterization needed	[153]
Candied jujube	Further characterization needed	2.25 g/L	-	Further characterization needed	[154]
Carob and Haricot bean	-	Up to 3.2 g/L	-	Optimized media for large-scale production	[142]
Maple syrup	Different possible applications	1.51 g/L	-	-	[145]
Pineapple and sugarcane juices	-	2.8 g/L	+30%	Low-cost substrate for large-scale industrial production.	[146]
Brewery waste	-	5.05 g/L	-	-	[155]
Olive mill wastewater and cheese whey	-	-	-	Basis for industrial scale-up	[138]
Figs waste	-	8.45 g/L	-	-	[15]
Bread waste	-	-	-	Low-cost substrate for large-scale industrial production.	[139]
Fruits peel	-	Up to 0.48 g/L	-	-	[140]
Pineapple peels and banana extracts	-	2 g/L	300%	Production suitable for future industrialization	[147]
Beet molasses, vinasse, and waste beer fermentation broth	-	Up to 5 g/L	-	-	[143]
Asparagus waste	Biomaterials	2.57 g/L	-	-	[148]
Domestic food waste	Packaging and biomedical applications	25 g/L	-	-	[115]
Waste apple pulp and stale bread	-	Up to 3.4 g/L	-	-	[149]

## 7. Bacterial Cellulose for Industrial Applications

### 7.1. Biomedical Application

BC is a renewable biomaterial with remarkable physical and chemical properties, such as non-toxicity, high biodegradability, cell immobilization, biocompatibility, and tunable chemical groups. These characteristics make BC highly suitable for application in tissue engineering [156], wound dressing [157], and drug delivery [4,158,159,160].

One of the main drawbacks of hydrogels is their high water absorption capacity, which reduces polymer network density and drastically weakens their mechanical strength. This limitation restricts their use in applications requiring high mechanical performance. To overcome this challenge, a potential solution is to incorporate fibrous reinforcements into hydrogel inks [161]. Hydrogels with self-assembling BC can be chemically modified, displaying augmented and tunable stiffness while guiding cell alignment. The BC nanofiber forms a 3D network, enhancing the mechanical strength of photo-cross-linkable hydrogels. It is possible to improve the mechanical strength of BC sheets by inducing nanofiber alignment or modifying nanofiber density through stretching and compression pretreatments [158,161].

BC 3D printing offers the potential for personalized constructs with improved biomechanical benefits and durability [23]. However, despite these advantages, BC sheets are challenging to use in conventional 3D printing [161]. Therefore, post-culture processing techniques, such as acidic hydrolysis, are needed to make BC sheets extrudable, enabling the fabrication of structures like cellularized constructs [162].

### 7.2. Sensors

BC has also proven useful in electronics, contributing to the production of flexible displays and various electronic components, including capacitors, flexible electrode materials, and biosensors [159]. BC exhibits an extraordinary specific tensile strength of approximately 852 MPa g^−1^ cm^3^, toughness, flexibility, and transparency, making it ideal for applications in flexible electronics [161].

Hydrogels have gained significant traction in flexible sensing applications due to their tunable modulus and excellent conformability to human skin. However, synthetic hydrogels face limitations as they may contain residuals of hazardous polymer monomers and reactants. Moreover, they lack biodegradability and do not align with the circular economy approach. To address these limitations, using environmentally friendly materials presents a viable solution. Hydrogels incorporating BC as fillers or substrates are emerging as strong candidates for the next generation of flexible sensors due to their abundant availability, outstanding biocompatibility, and superior mechanical properties [160].

Fiber-based resistive strain sensors have garnered significant attention for smart wearable devices due to their portability, flexibility, and ease of conformability. However, most existing fiber-based resistive strain sensors rely on metals and non-degradable polymers, making them environmentally unfriendly and mechanically weak. BC has emerged as a potential candidate for fabricating biodegradable conductive fibers. The nanostructure of BC, rich in hydroxyl groups, provides ample functional sites for doping or synthesizing conductive materials. Consequently, it is feasible to incorporate conductive nanomaterials into BC nanofibers and produce conductive macrofibers with high mechanical strength and biodegradability, ideal for strain sensors [37]. For example, robust and biodegradable conductive macro-fibers have been made by polymerizing p-toluene sulfonic acid-doped polypyrrole within BC nanofibers [37].

### 7.3. Other Applications of Bacterial Cellulose

In addition to the applications mentioned above, BC is used in food [45], cosmetics [36,159], and infrastructure industries [163].

BC is employed as a packaging material [164,165,166] and serves as a thickening, stabilizing, and emulsifying agent in the food industry. Since BC is mainly made up of cellulose, the Food and Drug Administration (FDA) classifies it as generally recognized as safe (GRAS) [36]. For instance, BC functions as a thickening and stabilizing agent, with studies showing that even a small amount of BC (1%wt) can improve the stability of olive oil-based emulsion, making it a valuable alternative to XG. BC also enhances the texture and mouthfeel of sauces [167] and contributes to smooth and uniform mixtures in products such as slow-melting ice creams [168] and low-fat baked foods [169].

In recent years, BC has also been used in the cosmetic sector. Its commercial potential has led to numerous patents covering various formulations and applications, including facial masks, moisturizers, skincare films, and lipsticks [170]. Facial masks are the most commonly patented and valued for their anti-hornification effect, high water absorption and retention, excellent wet strength, and outstanding antibacterial and antioxidant properties [171].

Finally, BC has been employed as a source of microfibers to reinforce soil matrices in construction fields. Studies have found that BC improves the soil’s physico-mechanical properties, enhancing mechanical properties and crack resistance. Moreover, BC incorporation in soil matrices reduces shrinkage, improving soil stability [172].

## 8. Bacterial Cellulose in the Fashion Sector

### 8.1. Environmental Stress Due to the Textile Sector

The textile industry depends heavily on industrial agriculture for cellulosic fibers, non-renewable petrochemicals for synthetic fibers, and chemically- and energy-intensive processes. It contributes 10% of global carbon emissions, 20% of global wastewater, and 35% of marine microplastic pollution [173]. Since 1990, synthetic fibers—manufactured from high-molecular-mass polymers—have gained significant market attraction, surpassing cotton. In 2019, synthetic fibers accounted for up to 63% of global fiber production, with polyester leading at 52% of the fiber market, followed by cotton at 23% [174].

The rapid expansion of clothing and footwear production is driven by fast fashion, a business model that offers consumers affordable and trend-focused products [175]. The growing population and fast fashion combination have led to significant increases in synthetic textile production. Between 2007 and 2015, global textile fiber production reached 92 million tons, with projections indicating an additional increase of 56 million tons by 2030.

The emerging eco-fashion industry aims to design garments with minimal environmental impacts, prioritizing environmental standards through optimized design, material, and production conditions [176]. For these reasons, the fashion and textile manufacturing industries seek innovative, renewable, and biodegradable raw materials. Bio-based hydrogels hold significant potential due to their renewability and biodegradability [177].

### 8.2. Environmental Stress Due to the Leather Industry

Increasing awareness of synthetic fibers’ environmental impact is pushing the fashion industry to commercialize alternatives to genuine leather. The tanning and finishing processes in leather production have a significant environmental footprint, with the apparel and footwear industries accounting for 8% of global greenhouse gas emissions. These emissions are projected to rise by over 60% by 2030 [178].

Environmental concerns regarding genuine leather have driven the development of synthetic alternatives made from PVC and PU, which have gained significant market traction over the past decades [5]. Although these synthetic materials eliminate the need for tanning chemicals, their production still depends on fossil-based resources [179,180].

The fashion industry has recently turned to vegan and bio-based alternatives to replace synthetic and genuine leather [181]. Various bio-based materials and technologies have been studied to create these alternatives [179,180]. To match genuine leather’s mechanical properties and durability, these materials are often reinforced with natural or synthetic fabrics. However, this reinforcement contributes to the environmental impact of these novel leather alternatives, as highlighted in Life Cycle assessment studies [181,182]. These innovative leather substitutes are produced with bio-based polymers [183,184], fungal mycelium [181,185,186], and BC [113,187]. Despite their promising sensory attributes, achieving the right balance between functionality, sustainability, and reduced environmental footprint remains challenging.

### 8.3. Bacterial Cellulose as a Textile and Leather Alternative

Cellulose has become a focal point in sustainable fashion due to its unique combination of compostability, versatile sourcing, and evolving production methods that address environmental concerns. Processing natural fibers like cotton and wool demands significant water usage and synthetic chemicals to meet modern consumer needs [6]. In contrast, BC introduces groundbreaking possibilities for circular production (Section 5) and exhibited a lower environmental impact than cotton, viscose, and lyocell in key environmental categories, such as land use and water depletion [188].

BC has become one of the most extensively studied bio-based materials among the next-generation alternatives to replace synthetic clothing and leather. In recent years, this biopolymer has been optimized to produce BC-based composite materials for fashion-related applications, including circular manufacturing processes utilizing food waste from industry (Table 2). One of the most significant challenges in shifting to bio-based alternative materials in the fashion industry, particularly for leather substitutes, is achieving mechanical properties comparable to genuine leather without reinforcing fabrics—one of the main contributors to pollutants during production. As a result, efforts are focused on achieving the desired mechanical properties without needing external meshes.

Research is increasingly focused on using discarded food and beverage waste as nutrients for BC production. For example, coconut water, a food waste byproduct, was used as a fermentation medium to biosynthesize BC. The obtained BC was crosslinked with oxidized edible oils and coffee grounds to create an alternative leather material. This circular manufacturing process enhanced the mechanical properties of BC, achieving a tensile strength of 82.14 MPa—twice that of genuine leather—without reinforcing fabrics [189]. Similarly, a combination of sour whey, apple juice, and brewer’s spent grains was explored as nutrient sources for BC production from kombucha tea, yielding 12.81 g/L of cellulose. The resulting bio-based composites, designed to mimic leather, incorporated glycerol, polycaprolactone (PCL), sunflower oil, polyvinyl alcohol (PVA), and plant leaf pulp. These materials exhibited increased hydrophobicity, flexibility, and mechanical stability, with a tensile strength of 1.7 MPa and an elongation at a breakpoint of 15% [187].

Further advancements have been made in optimizing BC-based materials for bio-related textiles and fashion applications. For example, a hydrogel produced with BC biosynthesized through kombucha tea fermentation was employed in the scalable manufacturing of t-shirt decorations and bracelets [190].

Moreover, a zero-waste BC production process was developed, allowing the cultivation of tailor-shaped BC sheets directly through kombucha tea fermentation. This method eliminated the need for cutting and reduced waste during garment production [191].

Incorporating other natural and synthetic materials has enhanced BC’s durability and functionality. For instance, the tensile strength of bio-leather was improved by integrating soy and mushroom proteins with glycerol, resulting in a material with a tensile strength of 647 N [192]. Similarly, hydrophobic BC materials were developed for use in the textiles and shoe industry by treating BC with commercial polymers such as polydimethylsiloxane, which increased the material’s water resistance, durability, and breathability [193].

Nanomaterials have also been explored to further enhance the properties of BC-based materials. BC was modified by incorporating gold nanoparticles, silver nanoparticles, and graphene oxide [194]. Graphene oxide augmented the mechanical properties, while silver nanoparticles imparted antibacterial features. Gold nanoparticles served as a bridge between BC, silver, and graphene oxide while providing the material with a desirable color.

Additionally, cellulose-based biocomposites were produced by blending BC with PU and polylactic acid (PLA), resulting in a hydrophobic surface and promoting elasticity through silane modification [113]. Dyeing techniques have also been explored from the perspective of circular manufacturing processes. Natural dyeing methods using coffee, ginger, and sappan wood were evaluated, with the dyes maintaining color stability for up to eight months of storage [127].

Research on BC-based materials for fashion-related products points to a more sustainable future by adopting bio-based alternatives to traditional and synthetic garments. Optimizing fermentation processes using food waste, alongside improving material performance, is vital for achieving successful large-scale production in an industrial scenario.

**Table 2:** Works employing bacterial cellulose-based biomaterial to produce fashion-related accessories.

BC Source	Alternative Material Features	Fashion Application	Scalability	Refs
-	Malleable, breathable, and water-impermeable BC-based nanocomposites impregnated with polydimethylsiloxane and perfluorocarbon	Textiles and shoes	Potential large-scale production	[193]
Kombucha tea fermentation	BC-derived hydrogel	Accessories and textile printings	Potential large-scale production	[190]
Kombucha tea fermentation	Dried BC sheets	Clothing material	-	[195]
Kombucha tea fermentation	BC-based composited with PVA, glycerol, PCL, and sunflower oil	Leather alternative	Scalable production	[187]
Kombucha tea fermentation	BC-polyurethane-polylactic acid composites	Textile, footwear, bags, and upholstery	-	[113]
Kombucha tea fermentation	BC-based materials enriched with soy and mushroom proteins	Leather alternative	-	[192]
Kombucha tea fermentation	Tailored-shaped BC	Textiles	-	[191]
Kombucha tea fermentation	BC-based composited with poly-vinyl-alcohol, glycerol, polycaprolactone, poly-lactic-acid	Textiles and shoes	-	[196]
Kombucha tea fermentation	BC-based composite with gold nanoparticles, silver nanoparticles, and graphene oxide	Leather alternative	-	[194]
Coconut water fermentation	Tanned BC sheets, oil-crosslinked and plasticized with glycerol	Leather alternative	To be improved to unlock industrial potential	[189]
Kombucha tea fermentation	Dried BC sheets dyed with coffee, ginger and sappan wood	-	Industrialization could be challenging	[127]
-	BC oil-plasticized	Leather alternative	-	[197]
-	Textile fibers produced with dry-jet BC	Textiles	Scaled process	[198]
Kombucha tea fermentation	Dried BC-sheets	Textiles	-	[199]
-	Melanated BC sheets	Textiles	Scalable BC	[200]

## 9. Conclusions and Future Perspectives

BC presents a highly promising alternative to synthetic and plant-based polymers due to its high purity, renewable nature, exceptional mechanical properties, and potential for sustainable and circular industrial applications. However, optimizing and standardizing fermentation parameters for cellulose production remains challenging for large-scale manufacturing. Using food waste in fermentation processes can enhance sustainability and promote circular production. Notably, in most reported approaches, the yield of BC using waste-derived nutrients has surpassed that of traditional media. Thus, upcycling waste for microbial fermentation aligns with a circular economy framework, contributing to sustainability and increased BC production.

Recent advances in biotechnology research and a deeper understanding of the mechanisms behind BC production have led to significant progress. For example, genetic manipulation and co-cultivation of microorganisms have enhanced production yields. Additionally, biotechnology techniques are pivotal in boosting BC yields and fine-tuning its physico-chemical properties. Combining circular and biotechnological approaches may support large-scale production, improve the tunability of biosynthesized BC properties, and offer environmental benefits. However, biotechnology tools are not fully empowered and therefore industrial scalability is still far from being achieved. The improvement of these technologies is mandatory for facilitating the industrial application of BC. Moreover, integrating BC in the textile sector could offer a sustainable solution to synthetic textiles and leather alternatives, helping to reduce fast fashion’s environmental impact. However, adopting BC-based materials for garments depends on improving production efficiency, reducing costs, and ensuring long-term durability.

In conclusion, while BC shows significant promise as a sustainable material, it is important to acknowledge that challenges related to the standardization of fermentation processes and the cost of cultivation media remain substantial hurdles. Addressing these issues will be decisive for the broad industrial adoption of BC. Therefore, future efforts focused on advancing biotechnological approaches and standardizing fermentation processes will be pivotal in scaling up BC production while maintaining high material quality.

## Data Availability

No new data were created or analyzed in this study. Data sharing is not applicable to this article.
